# Serum Metabolomics of Activity Energy Expenditure and its Relation to Metabolic Syndrome and Obesity

**DOI:** 10.1038/s41598-018-21585-6

**Published:** 2018-02-19

**Authors:** Marie S. A. Palmnäs, Karen A. Kopciuk, Rustem A. Shaykhutdinov, Paula J. Robson, Diane Mignault, Rémi Rabasa-Lhoret, Hans J. Vogel, Ilona Csizmadi

**Affiliations:** 1 University of Calgary, Department of Biochemistry and Molecular Biology, Calgary, T2N 1N4 Canada; 2 University of Calgary, Department of Biological Sciences, Calgary, T2N 1N4 Canada; 3 University of Calgary, Department of Oncology, Calgary, T2N 1N4 Canada; 4 University of Calgary, Department of Mathematics and Statistics, Calgary, T2N 1N4 Canada; 5 C-MORE, CancerControl Alberta, Alberta Health Services, Calgary, T5J 3H1 Canada; 6 Institut de Recherches Cliniques de Montréal, Montréal, H2W 1R7 Canada; 7 Université de Montréal, Département de Nutrition, Montréal, H3T 1J4 Canada; 8 University of Calgary, Community Health Sciences, Calgary, T2N 1N4 Canada

## Abstract

Modifiable lifestyle factors, including exercise and activity energy expenditure (AEE), may attenuate the unfavorable health effects of obesity, such as risk factors of metabolic syndrome (MetS). However, the underlying mechanisms are not clear. In this study we sought to investigate whether the metabolite profiles of MetS and adiposity assessed by body mass index (BMI) and central obesity are inversely correlated with AEE and physical activity. We studied 35 men and 47 women, aged 30–60 years, using doubly labeled water to derive AEE and the Sedentary Time and Activity Reporting Questionnaire (STAR-Q) to determine the time spent in moderate and vigorous physical activity. Proton nuclear magnetic resonance spectroscopy was used for serum metabolomics analysis. Serine and glycine were found in lower concentrations in participants with more MetS risk factors and greater adiposity. However, serine and glycine concentrations were higher with increasing activity measures. Metabolic pathway analysis and recent literature suggests that the lower serine and glycine concentrations in the overweight/obese state could be a consequence of serine entering de novo sphingolipid synthesis. Taken together, higher levels of AEE and physical activity may play a crucial part in improving metabolic health in men and women with and without MetS risk factors.

## Introduction

Obesity is a risk factor for the four most common chronic diseases worldwide: diabetes^1^, cardiovascular disease^2^, cancer^3^ and chronic respiratory disease such as asthma^4^. Together they contribute to substantial patient suffering, a serious economic cost to health care^5^ and lead to 30 million deaths annually^6^. Central (abdominal) obesity is of particular interest as it leads to the pathophysiology that results in metabolic syndrome (MetS)^7,8^, a cluster of risk factors (central obesity, hypertension, elevated fasting glucose and dyslipidemia) that, if untreated can lead to overt diabetes, cardiovascular disease and over the long-term to some cancers. The pathological development of MetS is characterized by an aberrant metabolism, in part consisting of a dysregulation in whole-body glucose and lipid metabolism, intra-organ lipid storage and systemic inflammation.

Since circulating metabolites are representative of systemic metabolism, they can reflect the metabolic health of an individual. Thus, metabolomics, the systematic study of a comprehensive set of metabolites in a biological compartment, has the potential to provide insight into the biological mechanisms underlying health and disease. To better understand the metabolic impact of obesity and its etiologic role in the development of diabetes and subsequent chronic diseases, attempts have been made to identify biomarkers using various ‘omics’ technologies, including metabolomics. Concentrations of branched chain amino acids (BCAA: leucine, isoleucine and valine) are the most extensively studied metabolic signatures of obesity and insulin signaling^9,10^ and increased plasma BCAA levels have been reported to be predictive of diabetes incidence years prior to diagnosis^11,12^. However, other metabolites may also play roles in the relation between obesity and obesity-related disorders.

The beneficial effects of physical activity as part of disease management and prevention have been clearly established for conditions such as type 2 diabetes, cardiovascular disease and certain types of cancer^13^. Yet, it is unclear through which mechanisms exercise and activity energy expenditure (AEE) attenuate the unfavorable effects of obesity, independent of weight reduction^14^. In this study we aimed to describe the metabolite profile associated with MetS and adiposity in relation to AEE and physical activity in weight stable men and women with and without MetS risk factors. Doubly labeled water (DLW), the gold standard methodology for assessing total energy expenditure^15,16^, was used to objectively assess AEE (AEE_DLW_) and physical activity level (PAL_DLW_). In addition, the Sedentary Time and Activity Reporting Questionnaire (STAR-Q) was used to determine the time spent in moderate and vigorous physical activity. We hypothesized that increased adiposity and the presence of MetS risk factors would lead to gender-specific metabolic signatures^17,18^. We further hypothesized that some of these metabolites would be inversely associated with AEE_DLW_, AEE/kg_DLW_, PAL_DLW_, moderate and/or vigorous physical activity, as exercise has previously been shown to influence the concentrations of circulating metabolites^19–21^.

## Results

### Data overview and characteristics of measured variables

Gender-stratification of the data was supported by the pronounced difference in the serum metabolome of men and women (R^2^ = 0.73, Q^2^ = 0.61) (Supplementary Fig. S1). Moreover, men had higher BMI, waist circumference, waist-to-hip ratio and body fat percentage compared to women (Supplementary Table S1) and presented with higher serum concentrations of BCAA and lower serum concentrations of serine and glycine, among other metabolites. No differences were seen for AEE_DLW_, AEE/kg_DLW_, PAL_DLW,_ moderate or vigorous physical activity. Information regarding all quantified metabolites can also be found in Supplementary Table S2.

### The serum metabolite profiles of MetS and correlations with measures of adiposity

Men and women were dichotomized into categories representing presence of 1–3 MetS risk factors (MetS_any_) and absence of MetS risk factors (MetS_zero_). Participants with MetS_any_ had significantly higher body measurements compared to their counterparts, and MetS_any_ men were more likely to be overweight (BMI > 25) whereas MetS_zero_ men were of normal weight (BMI 18.5–24.9) (Table 1). This is consistent with the known pathophysiology of MetS and its relation to adiposity^22^. There were no differences between groups in physical activity or DLW-derived measures of AEE.

**Table 1:** Evaluation of differences in variables for MetS_any_ compared to MetS_zero_ for women and men separately.

Variables	Women			Men		
	MetS_any_ Mean (SD)	MetS_zero_ Mean (SD)	p-value	MetS_any_ Mean (SD)	MetS_zero_ Mean (SD)	p-value
BMI (kg/m^2^)	24.6 (2.8)	21.6 (2.0)	<0.001	27.4 (2.5)	23.4 (1.9)	<0.0001
Body fat percentage (%)	33.3 (5.8)	25.2 (5.1)	<0.0001	24.8 (4.3)	15.6 (3.7)	<0.0001
Waist circumference (cm)	86.9 (6.8)	75.1 (4.5)	<0.0001	98.8 (8.1)	84.7 (5.2)	<0.001
Hip circumference (cm)	103.2 (6.0)	94.5 (5.4)	<0.0001	105.3 (4.3)	97.7 (3.7)	<0.001
Waist:hip ratio	0.84 (0.04)	0.79 (0.03)	<0.001	0.94 (0.06)	0.87 (0.04)	<0.001
AEE_DLW_ (Kcal)	1021.3 (460.0)	948.5 (438.5)	0.59	1163.0 (469.2)	1419.8 (772.8)	0.23
AEE/kg_DLW_ (Kcal/kg)	15.1 (6.8)	16.9 (7.6)	0.42	13.4 (5.6)	19.9 (10.7)	0.024
PAL_DLW_ (Kcal)	1.9 (0.4)	1.9 (0.4)	0.99	1.8 (0.3)	2.0 (0.5)	0.080
Moderate physical activity (hours/day)	1.5 (1.3)	1.6 (2.1)	0.81	2.2 (2.1)	0.9 (0.5)	0.065
Vigorous physical activity (hours/day)	0.54 (0.6)	0.55 (0.5)	0.96	0.45 (0.5)	0.82 (0.6)	0.061

Significance was determined by two-tailed t-test, where the Benjamini and Hochberg corrected significance level was p < 0.028. Abbreviations are as follows in alphabetical order: AEE_DLW_, activity energy expenditure; BMI, body mass index; MetS, metabolic syndrome; PAL_DLW_, physical activity level: SD, standard deviation.

**Figure 1:**
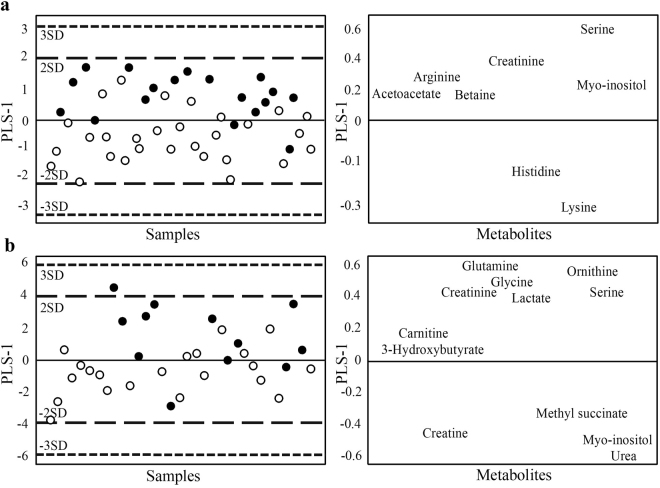
Supervised OPLS-DA score scatter plots and loadings plots showing the separation between MetS_any_ (circles) and MetS_zero_ (dots) for (**A**)women and (**B**) men. Every dot/circle represents one participant. The score scatter plot (left) and loading plot (right) are superimposable and indicate which (VIP > 1) metabolites associate with which MetS group in women (R^2^ = 0.42, Q^2^ = 0.30) and in men (R^2^ = 0.31, Q^2^ = 0.12), respectively.

The metabolite profiles of MetS were unique for both genders, with the exception of serine and creatinine, which were lower in both MetS_any_ men (R^2^ = 0.31, Q^2^ = 0.12) and MetS_any_ women (R^2^ = 0.42, Q^2^ = 0.30) compared with the respective MetS_zero_ groups (Fig. 1).

MetS_any_ men had higher serum metabolite concentrations of methyl succinate, creatine, myo-inositol and urea and lower concentrations of glutamine, ornithine, glycine, serine, creatinine, lactate, carnitine and 3-hydroxybutyrate compared with the MetS_zero_ group. Glycine and serine were also inversely correlated with all measures of adiposity, except that glycine was not associated with BMI class (VIP > 1, Supplementary Table S3**)**. The observed negative correlation between glycine and the waist-to-hip ratio, a measure thought to best represent male adiposity and fat distribution, reached bivariate statistical significance (r = −0.47, p < 0.01) in addition to the positive correlation found between glycine and vigorous physical activity (r = 0.48, p < 0.01). Other trends included higher concentrations of arginine, creatine, methyl succinate, pyruvate and tyrosine with increasing body measures in men (Table 2).

**Table 2:** Pearson correlation coefficients (r) and p-values (p) for significant (p < 0.05, VIP > 1) metabolites for each variable for women and men.

**Body fat percentage**	**r**	**p**	**BMI value**	**r**	**p**			
**Women**								
Serine	−0.45	0.0015	Serine	−0.36	0.012			
Creatine	0.31	0.034						
**Hip circumference**	**r**	**p**	**Waist circumference**	**r**	**p**	**Waist:Hip ratio**	**r**	**p**
Serine	−0.40	0.0049	Serine	−0.49	<0.001	Serine	−0.38	0.0090
Pyruvate	0.30	0.043	Myo-Inositol	−0.36	0.012	Myo-Inositol	−0.31	0.032
**AEE**_**DLW**_			**Strenuous PA**	**r**	**p**			
Myo-Inositol	−0.34	0.021	Glutamine	−0.37	0.011			
Acetoacetate	0.31	0.036	Creatine	−0.32	0.030			
			Phenylalanine	−0.32	0.030			
			Carnitine	−0.29	0.045			
**Men**								
Pyruvate	0.41	0.015	Tyrosine	0.48	0.013			
Creatine	0.37	0.026	Carnitine	0.37	0.028			
Arginine	0.36	0.036	3-Hydroxybutyrate	−0.36	0.032			
**Waist circumference**	**r**	**p**	**Waist:Hip ratio**	**r**	**p**			
Methyl succinate	0.40	0.017	Glycine	−0.47	0.0049			
Pyruvate	0.37	0.031	Arginine	0.46	0.0059			
Arginine	0.34	0.042	Pyruvate	0.36	0.033			
**AEE**_**DLW**_			**Moderate PA**	**r**	**p**	**Strenuous PA**	**r**	**p**
Carnitine	0.42	0.011	Carnitine	0.41	0.013	Glycine	0.48	0.024
Lactate	0.42	0.012	Lactate	0.37	0.017	Taurine	−0.35	0.043
Lysine	0.40	0.018	Methionine	0.32	0.048	Acetate	0.35	0.048

Abbreviations are as follows in alphabetical order: AEE_DLW_, activity energy expenditure; BMI, body mass index; PA, physical activity.

MetS_any_ women had higher levels of histidine and lysine and lower levels of serine, creatinine, myo-inositol, arginine, acetoacetate and betaine compared with MetS_zero_ women. The lower serine concentrations in MetS_any_ women were also significant with univariate analysis (p < 0.001). Serine was furthermore found to be lower with increasing values of all body measurements in women and as the number of MetS risk factors increased (Supplementary Table S4). Of note, overweight and increasing MetS risk factors were associated with lower glycine concentrations in women (VIP > 1, Supplementary Table S4).

### MetS-associated metabolites are inversely associated with measures of AEE_DLW_ and physical activity in men and women

**Figure 2:**
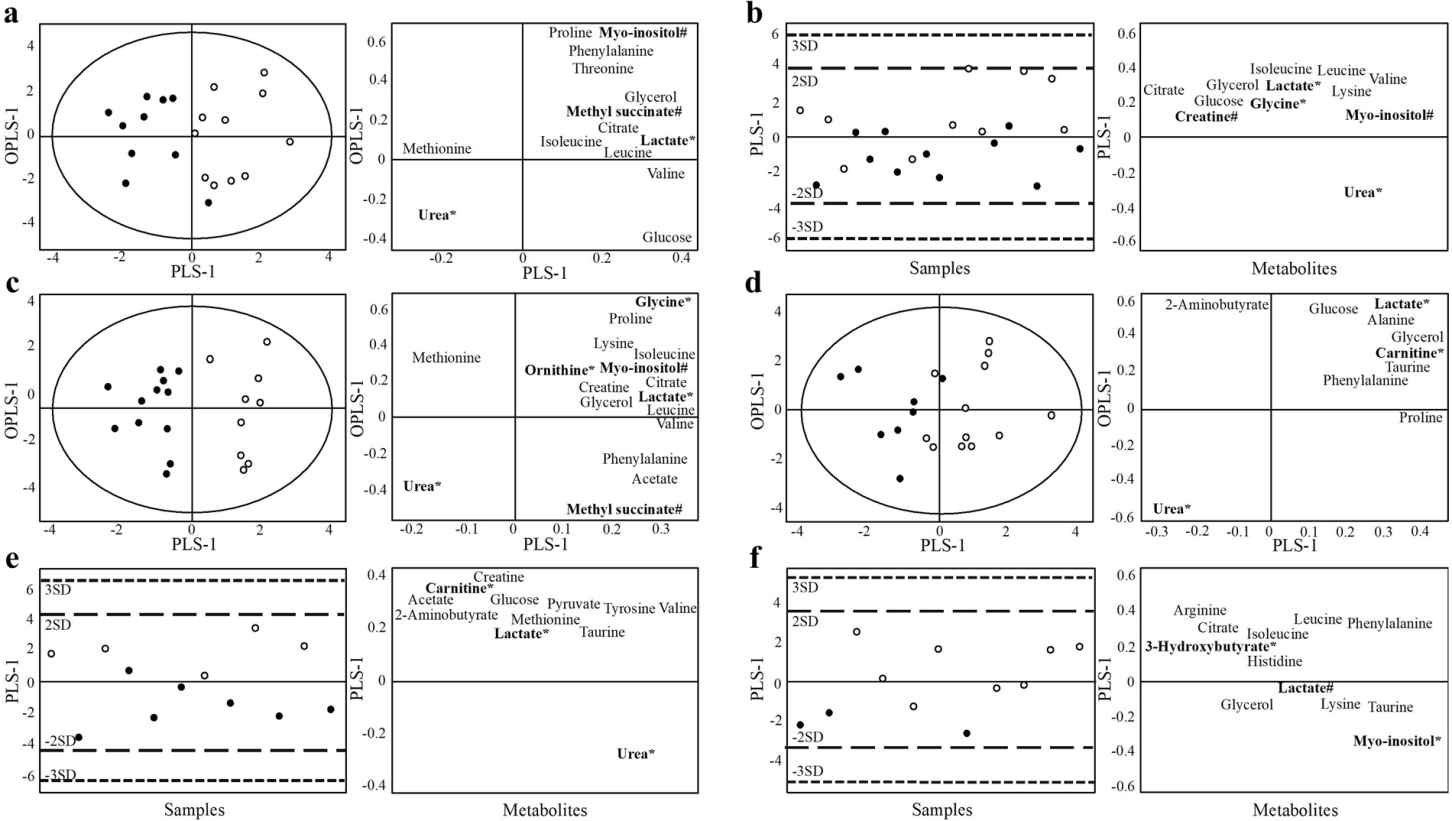
Supervised OPLS-DA score scatter plots and loadings plots for men, showing AEE_DLW_ and physical activity variables stratified for MetS_any_ (**A–D**) and MetS_zero_ (**E–F**). Every dot/circle represents one participant, with circles indicating participants with the highest 50% (circles) and the lowest 50% (dots) of each variable. The score scatter plots (left) and loading plots (right) are superimposable and show (VIP > 1) metabolites. Only variables that resulted in models are shown. (**A**) AEE_DLW_ (R^2^ = 0.67, Q^2^ = 0.36), (**B**) AEE/kg_DLW_ (R^2^ = 0.34, Q^2^ = 0.24), (**C**) PAL_DLW_ (R^2^ = 0.86, Q^2^ = 0.46), (**D**) moderate physical activity (R^2^ = 0.38, Q^2^ = 0.21) for MetS_any_ and (**E**) moderate physical activity (R^2^ = 0.62, Q^2^ = 0.51) and (**F**) vigorous physical activity (R^2^ = 0.57, Q^2^ = 0.25) for MetS_zero_.

Next, we were interested in examining whether MetS-associated metabolites were inversely related with AEE_DLW_, AEE/kg_DLW_, PAL_DLW_, moderate or vigorous physical activity in comparison to MetS using multivariate analysis. The highest 50% (high groups) and lowest 50% (low groups) of each activity measurement were compared within the respective MetS_any_ and MetS_zero_ groups of men and women. The supervised analysis showed MetS_any_ men to have higher concentrations of glycine and lactate with the higher levels of PAL_DLW_ (R^2^ = 0.86, Q^2^ = 0.46) and AEE/kg_DLW_ (R^2^ = 0.34, Q^2^ = 0.24) compared with the low PAL_DLW_ and AEE/kg_DLW_ groups (Fig. 2). Lactate was furthermore positively correlated with AEE_DLW_ (R^2^ = 0.67, Q^2^ = 0.36) and moderate physical activity (R^2^ = 0.38, Q^2^ = 0.21) in MetS_any_ men and with moderate (R^2^ = 0.62, Q^2^ = 0.51) and vigorous physical activity (R^2^ = 0.57, Q^2^ = 0.25) in MetS_zero_ men. Of note, none of these variables were correlated with body measures in MetS_any_ or MetS_zero_ men as assessed by bivariate Pearson correlation on the continuous values.

**Figure 3:**
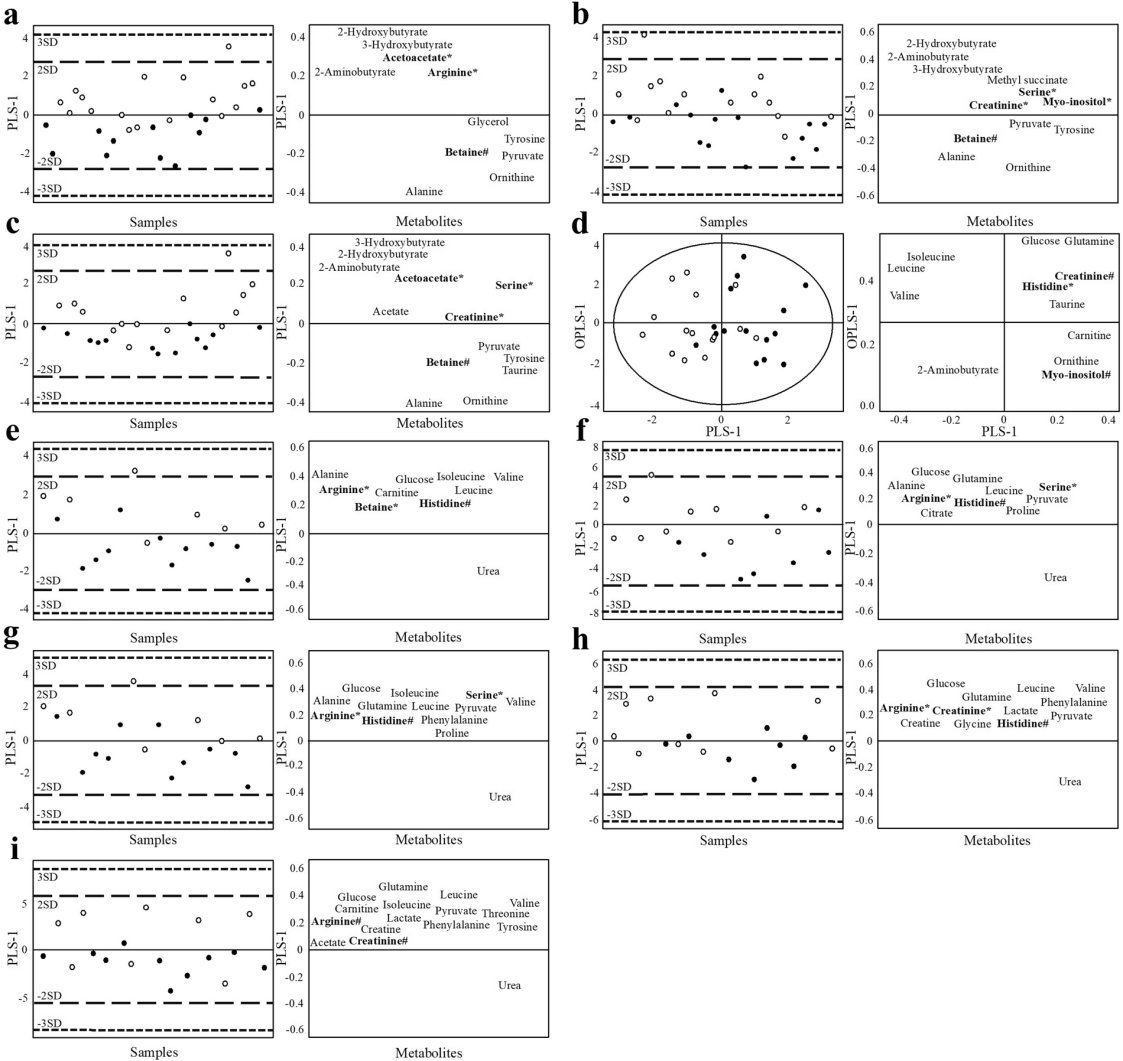
Supervised OPLS-DA score scatter plots and loadings plots for women, showing AEE_DLW_ and physical activity variables stratified for MetS_any_ (**A**–**D**) and MetS_zero_ (**E**–**I**). Every dot/circle represents one participant, with circles indicating participants with the highest 50% (circles) and the lowest 50% (dots) of each variable. The score scatter plots (left) and loading plots (right) are superimposable and show VIP > 1 metabolites. Only variables that resulted in models could be shown. The variables presented include (**A**) AEE_DLW_ (R^2^ = 0.45, Q^2^ = 0.27), (**B**) AEE/kg_DLW_ (R^2^ = 0.33, Q^2^ = 0.11), (**C**) PAL_DLW_ (R^2^ = 0.44, Q^2^ = 0.26), (**D**) vigorous physical activity (R^2^ = 0.43, Q^2^ = 0.22), for MetS_any_ and (**E**) AEE_DLW_ (R^2^ = 0.43, Q^2^ = 0.37), (**F**) AEE/kg_DLW_ (R^2^ = 0.32, Q^2^ = 0.21), (**G**) PAL_DLW_ (R^2^ = 0.40, Q^2^ = 0.33), (**H**) moderate physical activity (R^2^ = 0.28, Q^2^ = 0.14) and (**I**) vigorous physical activity (R^2^ = 0.28, Q^2^ = 0.16) for MetS_zero_.

For MetS_zero_ women, serine and arginine were found to be higher with high levels of AEE/kg_DLW_ (R^2^ = 0.32, Q^2^ = 0.21) and PAL_DLW_ (R^2^ = 0.40, Q^2^ = 0.33) compared with low levels in MetS_zero_ women (Fig. 3). Arginine and betaine were also higher with AEE_DLW_ (R^2^ = 0.43, Q^2^ = 0.37). For MetS_any_ women, AEE_DLW_, AEE/kg_DLW_ and PAL_DLW_ were positively correlated with several metabolites that had been found to be lower in the presence of MetS risk factors (Fig. 3). For example, higher levels of AEE and AEE/kg were associated with higher serum concentrations of acetoacetate and arginine as well as creatinine, myo-inositol and serine, respectably, compared with lower levels (Fig. 3). The high PAL_DLW_ group had higher concentrations of acetoacetate, creatinine and serine compared to the low PAL_DLW_ group. Notably, women in the MetS_zero_ and MetS_any_ groups of higher levels of physical activity, AEE_DLW_, AEE/kg_DLW_ and PAL_DLW_ had lower body measures overall (Supplementary Table S4). Thus, the impact of the higher activity measurements on the serum metabolome cannot be defined as independent of the lower body measures. Yet, these patterns are consistent with known correlations of lower body weight with higher AEE and physical activity^23,24^.

**Figure 4:**
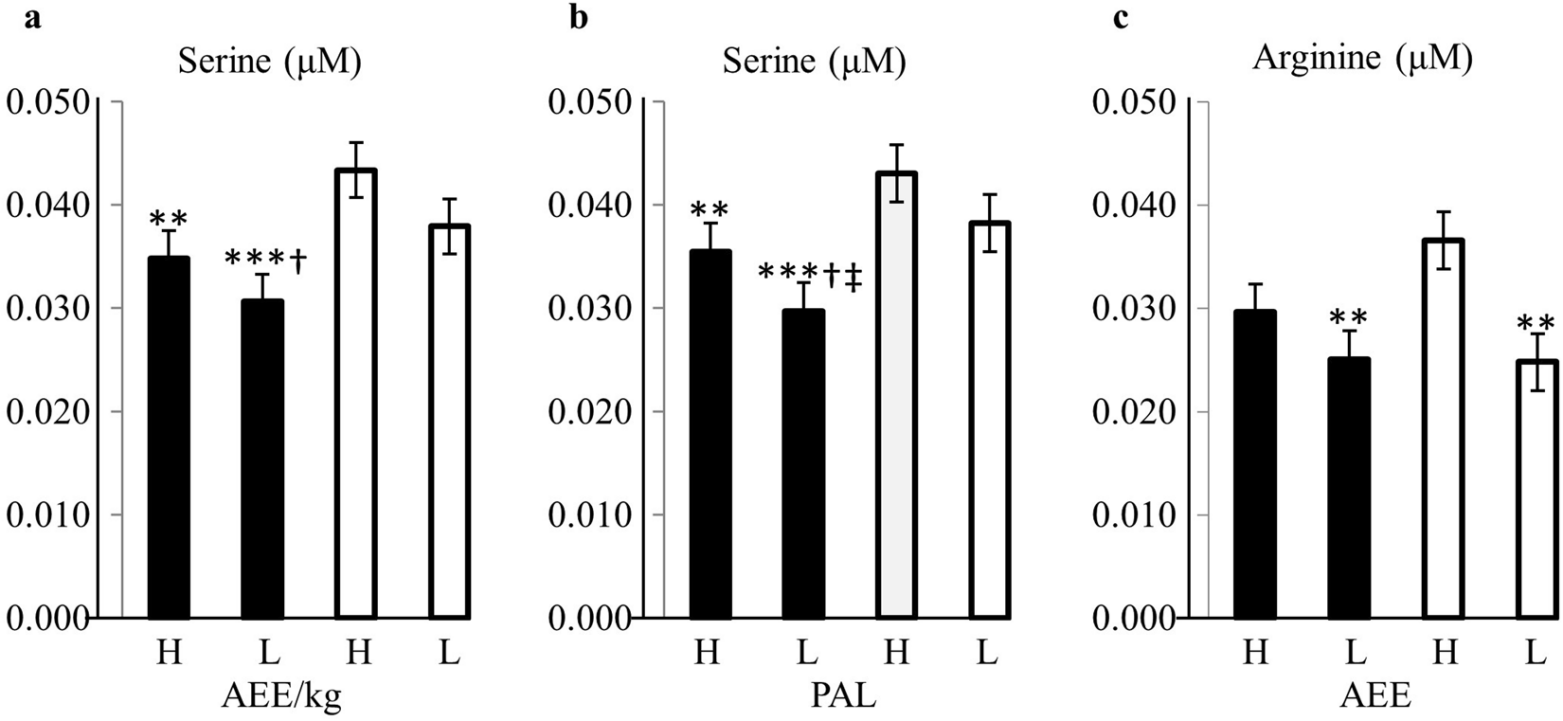
Serum serine and arginine concentrations for MetS_any_ (black bars) and MetS_zero_ women (white bars) with high (H) or low (L) levels of AEE/kg_DLW_ and PAL_DLW_, and AEE_DLW_ respectively. Significant difference compared to MetS_zero_-H (**p < 0.01 ***p < 0.001), MetS_zero_-L (†p < 0.05) and MetS_any_-H (‡p < 0.05) is indicated. Serum concentrations were normalized to the total sum for each sample to assure normal distribution and comparability across samples.

Finally, since serine and arginine had correlated with DLW measurements in both MetS_any_ and MetS_zero_ women, we compared the serum concentrations for the 4 groups of high and low activity measurements of MetS_any_ and MetS_zero_ women, respectively (Fig. 4). For serine there were 3 main findings; 1) MetS_zero_ women with high PAL_DLW_ and AEE/kg_DLW_ had the highest concentration of serine 2) for MetS_any_ women, higher PAL_DLW_, but not AEE/kg_DLW_, was associated with higher serine concentrations and 3) MetS_any_ women with high PAL_DLW_ and AEE/kg_DLW_ have comparable serine concentrations to the MetS_zero_ women with low PAL_DLW_ and AEE/kg_DLW_. Arginine concentrations were significantly higher in MetS_zero_ women with high AEE_DLW_ compared to both the MetS_any_ and the MetS_zero_ women with low AEE_DLW_ (p < 0.01).

### Sphingolipid metabolism potential pathway for MetS

To gain further insight into the biological mechanisms underlying the reported metabolite findings, pathway analysis was performed on all analyzed metabolites. Two pathways were significant when comparing MetS_any_ to MetS_zero_ women, both involving serine; ‘sphingolipid metabolism’ (serine, p < 0.001) and ‘methane metabolism’ (serine and glycine, p < 0.05) (Supplementary Table S6**)**.

No statistically significant pathways could be identified for MetS for men or for BMI, waist circumference, AEE_DLW,_ PAL_DLW_ or physical activity measurements in either gender after correcting for false discovery rate. However, there were pathways that had significant unadjusted p-values. For example, waist circumference associated with multiple pathways involving serine and/or glycine in men, including sphingolipid metabolism, glycine, serine and threonine metabolism, methane metabolism, sulfur metabolism, cysteine and methionine metabolism and cyanoamino metabolism (p_unadjusted_ < 0.05, data not shown).

## Discussion

Although much is known about the physiological impact of exercise on immediate fuel utilization, body coordination, cognitive function and cardiorespiratory fitness, there is a large gap in the understanding of how these and other changes are linked to long-term disease prevention and amelioration^14^. Metabolomics and other “omics” approaches can potentially provide insight to the mechanisms linking exercise and health.

In this study we describe the serum metabolite signatures associated with adiposity in relation to AEE_DLW_, AEE/kg_DLW_, PAL_DLW_ and moderate and vigorous physical activity in men and women with or without MetS risk factors. The most consistent metabolomics trend consisted of the lower serum concentrations of serine in the presence of MetS risk factors and greater adiposity in both men and women. Glycine was also found at lower concentrations with a higher number of MetS risk factors in both genders and with higher adiposity in men. In contrast, DLW-assessed measurements i.e. AEE/kg_DLW_ and PAL_DLW_, but not self-reported moderate or vigorous physical activity, were associated with higher concentration of serine in MetS_any_ women and glycine in MetS_any_ men. Interestingly, higher PAL_DLW_ levels in MetS_any_ women were associated with serine levels comparable to the less active MetS_zero_ women. This correlation was partly dependent on the positive correlation between BMI and PAL_DLW_ in the high PAL_DLW_ MetS_any_ group. We speculated that this may be an indication of leanness since 1) the BMI was lower in the high PAL_DLW_ group compared to the low group, with most women in the high PAL group having a BMI within the normal range and since 2) BMI was inversely correlated with serine levels in women in the low PAL_DLW_ and AEE/kg_DLW_ groups, yet without statistical significance. The highest serine concentrations were seen for MetS_zero_ women with the highest PAL_DLW_, attesting to the importance of engaging in physical activity even for normal weight and healthy women. Our results also suggest that high PAL may mitigate the impact of adiposity and MetS risk factors on the serum metabolome in women. Of note, exercise has also been shown by others to influence the human serum and plasma metabolite profiles^19–21^. This is supported in animal models, showing exercise to mitigate the metabolite profile associating with diet-induced obesity^25^ and diabetes^26^.

Similar to our findings, others have found decreasing serine and glycine concentrations with obesity, insulin resistance, diabetes and MetS risk factors^27–36^. For example, glycine concentrations have previously been shown to be linked with whole body glucose uptake and thus insulin responsiveness, as assessed by hyperinsulinemic-euglycemic glucose clamps^29^. Moreover, lower serine and glycine levels were reported to be instrumental in differentiating between insulin sensitive, insulin resistant and diabetic adults^29^. Serine and glycine have also been reported to positively correlate with insulin action, as assessed by repeated glucose tolerance tests in overweight and obese sedentary men and women^32^. In addition, serine has been shown to be correlated with impairments in fasting glycemia in type 2 diabetic adults, while fasting concentrations of glycine have been reported to be lower in diabetic compared to normoglycemic adults^31^. A metabolic signature consisting of glycine in combination with phenylalanine, hexose, sphingomyelin 16:1 and phosphatidylcholines has furthermore been able to predict diabetes with high accuracy 7 years prior to clinical manifestation^36^. Finally, Batch *et al*. reported that the combination of reduced serine, glycine and ornithine concentrations had the potential to distinguish between metabolically well and unwell (>2 MetS risk factors) adults, after adjusting for BMI^30^. Importantly, none of these studies assessed physical activity or objective measures of AEE to determine their relation to these metabolites.

The pathway analysis suggested sphingolipid metabolism as a potential underlying mechanism to the detected inverse correlations between serine and MetS risk factors. This finding is supported by recent reports indicating that obesity, insulin resistance and metabolic syndrome are associated with lower concentrations of serine^28,29,32,37^ and glycine^27,29–31,38^ (substrates) and higher concentrations of sphingolipid species^39–64^ (products) in humans. Interestingly, the opposite trend was seen for physical activity^65–74^, with some studies reporting conflicting results^28,45,67^. Dubé *et al*. also showed that exercise, but not dietary restriction, reduced ceramide and sphingosine levels, while both approaches improved on insulin sensitivity^65^. It is thus possible that serine is used as a precursor for sphingolipids in the obese state, leading to the accumulation of the bioactive lipid ceramide in insulin sensitive tissues, such as muscle and liver, where it can contribute to the development of insulin resistance^75^. In contrast, serine may be spared with physical activity, perhaps by the downregulation of serine palmitoyltransferase^76^, the enzyme that catalyzes the first step of de novo sphingolipid synthesis utilizing serine and palmitoyl-CoA to produce 3-ketosphinganine. An exercise intervention study for participants with and without MetS investigating de novo sphingolipid metabolism with isotope labeled precursors and liquid chromatography mass spectrometry metabolomics could complement the present work. However, it is also possible for circulating serine concentrations to be dependent on other factors including diet, protein and phospholipid degradation or synthesis from 3-phosphoglycerate^77^.

Although serine and glycine were prominent features of this study, other metabolites may also play a crucial part in adiposity and MetS. Among the key findings were the lower levels of arginine with MetS risk factors in women and the higher levels with AEE/kg_DLW_ and PAL_DLW_ in both MetS_any_ and MetS_zero_ women. Arginine has multiple functions related to glucose and insulin concentrations; it is a gluconeogenic amino acid and has the ability to activate AMPK and mTOR, resulting in higher insulin secretion and glucose uptake^77^. However, arginine levels were positively correlated with body measures in men. Furthermore, creatinine was found to be lower in MetS_any_ men and women and to be positively correlated to AEE/kg_DLW_, PAL_DLW_ and moderate physical activity in MetS_any_ women as well as vigorous physical activity in MetS_zero_ women. Creatinine is a breakdown product of muscular creatine phosphate, and may thus reflect the higher levels of physical activity in these subjects. Finally, lactate concentrations were lower in the presence of MetS risk factors in men and higher with PAL_DLW_, AEE_DLW_ and moderate physical activity in MetS_any_ men and with moderate and vigorous physical activity in MetS_zero_ men. Concentrations of lactate are best known to increase during physical activity and to return to normal after approximately 30 minutes of active or passive recovery^78^. Lactate levels are also commonly found to be higher as a consequence of cancer and critical illness, however none of the participants in the present study had any chronic or acute illnesses. Interestingly, BCAA did not contribute significantly to the metabolite profiles of MetS or adiposity in our work, with the exception that BCAA concentrations were higher in men (who had significantly higher BMI, waist circumference and waist-to-hip ratio) compared to women. These results may reflect the natural variation between populations and habits in free-living humans, as BCAA concentrations have been reported to be influenced by factors such as BMI, diabetes and diet (proportion of fat and carbohydrates)^12,29^.

The limitations of the study need to be addressed. The relatively small sample size and lower proportion of male participants restricted statistical analyses and while creating subgroups increased the homogeneity of the groups it also decreased the power to detect true associations. For this reason, our findings need to be validated in larger well-controlled studies that investigate other possible metabolic pathways of interest. Studies containing 25–30 participants per group should suffice to detect key metabolites with modest differences in abundance levels between groups^79,80^. Another limitation included the reliance on an estimation of RMR (resting metabolic rate) using an equation based on gender, age and anthropometric measures, rather than an actual measure using indirect calorimetry, which may have resulted in reducing the precision of the AEE_DLW_ estimation (see Equation 1). As well, moderate and vigorous physical activity levels were estimated from self-report, known to be associated with measurement error. We have previously shown, however, that there is substantial agreement between vigorous physical activity estimated from the STAR-Q and prospectively collected 7-day activity diaries indicating that the estimate may be informative for the ranking of subjects according to vigorous physical activity levels^81^. On the other hand, a major strength of our study includes the use of DLW, the gold standard for TEE measurement, which allowed for objective estimations of AEE_DLW_, AEE/kg_DLW_ and PAL_DLW_. To our knowledge this is the first study to examine the relation between DLW-derived AEE_DLW_ and PAL_DLW_ estimates and metabolites in subjects with and without risk factors of MetS. This study also included anthropometric measures that were assessed by trained staff rather than having to rely on participant self-report. The multiple measures allowed for the investigation of the effect of site-specific adiposity with respect to certain metabolite profiles. Notably, AEE_DLW_ and BMI in men were positively correlated reaching statistical significance, potentially as a consequence of the higher ‘energy cost’ of movement with excess body mass^82^ as the men in the present study were overweight on average. Standardizing AEE_DLW_ to bodyweight partly reduced this association and AEE/kg_DLW_ was negatively correlated to waist circumference.

In conclusion, we observed for the first time that higher levels of DLW- derived PAL and AEE measures were inversely correlated with MetS and adiposity-associated metabolites. While not conclusive, our findings provide evidence to support the promising potential effects of physical activity and overall energy expenditure in mitigating the negative effects of obesity on metabolism. We suggest that serine and glycine, through the involvement in de novo synthesis of sphingolipids such as ceramides may play an important part in the development of metabolic disease from obesity. To the best of our knowledge, this is the first study to examine metabolite profiles associated with DLW-derived AEE and PAL in humans.

## Methods

### Study design and ethical approval

This study was comprised of a convenience sample of volunteer participants who were recruited to a validation study designed to examine the measurement properties of the STAR-Q^83^. The study design, recruitment and data collection procedures, excluding the metabolomics procedures, have previously been described^81^.

Of 102 participants in the STAR-Q validation study^81^, 82 (35 men and 47 women) provided overnight fasting blood samples from which quantitative metabolomics data (see below) were generated. Recruitment to the original study was based on the following inclusion criteria: 1) 30–60 years of age; 2) living in the Calgary, Alberta, Canada area; 3) weight stability (≤2.5 kilogram weight change for at least 3 months) and; 4) body mass index (BMI) ≤35. Pregnant or breastfeeding women, participants with metabolic disorders (e.g., diabetes and thyroid dysfunction) or individuals taking medications affecting water balance were not eligible for the study. All participants provided informed consent as well as detailed reports of their medical history. Ethical approval for the study was obtained from the Alberta Cancer Research Ethics Committee of Alberta Health Services and the Conjoint Health Research Ethics Board of the University of Calgary, Calgary, Alberta, Canada. All methods were performed in accordance with relevant guidelines and regulations.

### AEE_DLW_, PAL_DLW_ and physical activity measures

Doubly labeled water (DLW: deuterium and oxygen-18 (^18^O)) was used to derive total energy expenditure (TEE_DLW_). In brief, saliva and urine samples were obtained after an overnight fast for the determination of the background isotope levels (day 0). Participants were then orally administered 0.18 g 99 atom percent deuterium and 2.5 g 10 atom percent ^18^O per kilogram of estimated total body water. Post-dose saliva samples were collected at 3 and 4 hours and second-void urine samples were collected on days 1, 8 and 14. The samples were batch-analyzed in duplicate using an Isoprime Stable Isotope Ratio Mass Spectrometer (Isoprime Ltd., Cheadle Hulme, United Kingdom) to measure the decline in isotope enrichment. TEE_DLW_ was calculated according to the method of Racette *et al*.^84^ using a modified Wier equation and an assumed respiratory quotient of 0.85. AEE_DLW_ (and in extension AEE_DLW_/kg) was estimated from TEE_DLW_ using **Equation 1** below where resting metabolic rate (RMR) was estimated using the Schofield equation^85^ and hours of sleep as reported in the STAR-Q:

### AEE_DLW_ = 0.9TEE_DLW_ – ((RMR/24) × (24 - hrs of sleep)) + (0.9(RMR/24) × hrs of sleep)

Activities were self-reported for the previous month using the self-administered STAR-Q completed on day 14 of the DLW protocol. All activities were assigned activity codes and values for metabolic equivalents of task were obtained from the Compendium of Physical Activities^86,87^. Estimates of time spent in moderate (3.0–6.0 metabolic equivalents of task) and vigorous intensity physical activity (>6.0 metabolic equivalents of task) were then derived^81^. The physical activity level (PAL_DLW_) was estimated as the ratio of TEE_DLW_ to RMR.

### MetS risk factors and anthropometric measurements

MetS is defined as a cluster of risk factors comprising high blood pressure (systolic ≥ 130 mm Hg, diastolic ≥ 85 mm Hg), abdominal obesity (men ≥ 102 cm, women ≥ 88 cm)^88,89^, raised fasting glucose (≥ 5.6 mmol/l) and dyslipidemia, defined as low HDL (men < 1.0 mmol/l, women < 1.3 mmol/l) and high triglycerides (≥ 1.7 mmol/l) or alternatively, medication for any of the mentioned conditions^7^. Prescription drug use was ascertained at the study center visit at which time participants were requested to bring in medication containers. Triglycerides, HDL cholesterol, total cholesterol and fasting glucose were determined at the Calgary Laboratory Services using established protocols and fasting whole blood.

Anthropometric measures and blood pressure were determined at the study center by a certified exercise physiologist. Waist circumference was measured midway between the iliac crest and the lowest rib, as per Canadian guidelines^90^. Both waist and hip circumference were recorded to the nearest 0.5 cm. Weight and height were measured to nearest 0.1 kg and 0.1 cm, respectively, for BMI calculations (kg/m^2^). BMI was analyzed as a continuous and a binary variable that was dichotomized to represent healthy body weight (BMI < 24.9 kg/m^2^) and overweight and obese categories (BMI ≥ 25.0 kg/m^2^). Body fat percentage was determined using the TBF-310 Tanita Body Composition Analyzer and Scale (Tanita Corporation of America, Preston, USA). Blood pressure was assessed following the recommendations by the Canadian Hypertension Educational Program^91^.

### Serum metabolomics analysis using proton nuclear magnetic resonance spectroscopy

Whole blood was collected in Red Top vacutainers following a 10 hour fast, processed by centrifuge to yield serum and stored in aliquots of 0.5 mL cryovials in −80 °C until further analysis. At the time of analysis, samples were thawed on ice and larger molecules (e.g., proteins and lipid assemblies) were removed using 3-kDa Nanosep centrifugal filters and analyzed as previously described^92^. In brief, the filtrate volume was brought up to 650 μL, using a DSS-containing sodium phosphate buffer (130 μL, 0.5 M), the antibacterial compound sodium azide (10 μL, 1 M) and deuterium oxide. In addition, pH was adjusted to 7 ± 0.01 using sodium hydroxide and hydrochloride. Proton nuclear magnetic resonance spectroscopy (^1^H NMR) was used to acquire 1-dimensional spectra using a standard pulse program (prnoesy1d) on a Bruker Avance 600 spectrometer (600.22 MHz, 5 mm TXI Probe) at 298 °K. All samples were analyzed in automatic mode using Bruker NMRCase sample changer after shimming the first sample of each batch. Each sample was then processed using Chenomx NMR Suite 7.5 software (Chenomx Inc., Edmonton, Canada), comprised of line broadening (0.5 Hz), baseline and phase correction, water region deletion, shimming and concentration calibration. Quantitative metabolic profiling was performed using the NMR Suite profiling module employing the Chenomx library assisted by the human metabolome database (www.hmdb.ca) as well as 2-dimensional heteronuclear single quantum coherence spectroscopy (^1^H,^13^C HSQC) and total correlation spectroscopy (^1^H,^1^H TOCSY). All samples were coded with sample ID and prepared, analyzed and profiled in a randomized order.

### Statistical analysis

Serum metabolites and study variables were analyzed by univariate (two-tailed t-test assuming unequal variance), bivariate (Pearson correlation analysis) and multivariate statistical analysis (as described below). Statistical significance was defined as a p-value < 0.05 for uni- and bivariate statistical analysis and a variable importance in the projection-value (VIP) > 1.0, for the multivariate statistical analysis. Univariate and bi-variate p-values were corrected for false discovery rate (FDR) according to Benjamini and Hochberg^93^.

The analysis of the metabolomics data is based on work by Goodacre *et al*.^94^, and their standards for analysis. In brief, sample normalization of the metabolomics data was performed to ensure Gaussian distribution. The measured concentration of each metabolite in each sample was divided with the total concentration sum of that sample. Glucose, glycerol and lactate, which consistently had the highest concentrations in the samples, were excluded from the total sum in order to not dominate the normalization procedure. The normalized ^1^H NMR data was then used for the uni- and bivariate (Pearson correlation) statistical analysis using Microsoft Excel and imported into the SIMCA-P + software (version 12.01, Umetrics AB, Umeå, Sweden) for multivariate statistical analysis. In brief, for the multivariate analysis, data were further mean centered and scaled using unit variance scaling and statistical models were created based on 7-fold cross-validation. Unsupervised principal component analysis (PCA) was conducted to identify possible groupings of the data and to identify outliers, defined as samples outside the default 95 percent confidence interval. Outliers were excluded when creating supervised models. Only the models that were based on the most influential metabolites (VIP > 1) are shown. Partial least squares regression analysis (PLS) was applied for continuous Y-variables whereas PLS discriminant analysis (PLS-DA) and orthogonal PLS-DA (OPLS-DA) were applied for qualitative Y-variables with more than three classes (e.g., the number of MetS risk factors, MetS_0-3_) and two classes (e.g., BMI class and MetS_any_ vs Mets_zero_), respectively. All models were evaluated based on R^2^ and Q^2^ values, representing the explained variance and the predictive ability of the models, respectively. Multivariate statistical models are generally believed to not be over-fit if the R^2^Y and the Q^2^ values are within <0.3 of each other^80,95^. Analyses were carried out separately in men and women, and further stratified by MetS when indicated i.e., MetS_any_ (1–3 MetS risk factors) and MetS_zero_ (0 MetS risk factors).

### Metabolic pathway analysis

Metaboanalyst (www.metaboanalyst.ca) was used for the pathway analysis. The normalized data were imported and scaled and mean centered and the analysis specified for humans. Pathways were evaluated based on p-values corrected for false discovery rate^93^ with p < 0.05 indicating statistical significance.

### Data availability

The datasets generated during and/or analyzed during the current study are available from the corresponding author on reasonable request.

## Electronic supplementary material

Supplementary Information
