# Supplementary material for: Serum Metabolomics of Activity Energy Expenditure and its Relation to Metabolic Syndrome and Obesity
Source: Sci Rep. 2018 Feb 19;8:3308. doi: 10.1038/s41598-018-21585-6 (PMC5818610; doi:10.1038/s41598-018-21585-6)
Supplement: Supplementary file 1 — Supplementary Information [file 41598_2018_21585_MOESM1_ESM.pdf]

Supplementary Information

Serum Metabolomics of Activity Energy Expenditure and its Relation to Metabolic Syndrome and Obesity

Marie SA Palmnäs[1,2], Karen Kopciuk[3,4,], Rustem A Shaykhutdinov[2], Paula J Robson[5], Diane Mignault[6,], Rémi Rabasa-Lhoret[6,7], Hans J Vogel[1,2], Ilona Csizmadi[5,8*]*

[1] University of Calgary, Department of Biochemistry and Molecular Biology, Calgary, T2N 1N4, Canada, [2]University of Calgary, Department of Biological Sciences, Calgary, T2N 1N4, Canada, [3] University of Calgary, Department of Oncology, Calgary T2N 1N4, Canada, [4] University of Calgary, Department of Mathematics and Statistics, Calgary T2N 1N4, Canada [5]C-MORE, CancerControl Alberta, Alberta Health Services, Calgary, T5J 3H1, Canada, [6] Institut de Recherches Cliniques de Montréal, Montréal, H2W 1R7, Canada [7] Université de Montréal, Département de Nutrition, Montréal, H3T 1J4, Canada [8] University of Calgary, Community Health Sciences, Calgary, T2N 1N4, Canada.

TABLE OF CONTENT

Supplementary Figure S1 and legends	3
Supplementary Table S1 and legends	4-5
Supplementary Table S2 and legends	6-7
Supplementary Table S3 and legends	8-9
Supplementary Table S4 and legends	10-11
Supplementary Table S5 and legends	12
Supplementary Table S6 and legends	13-16

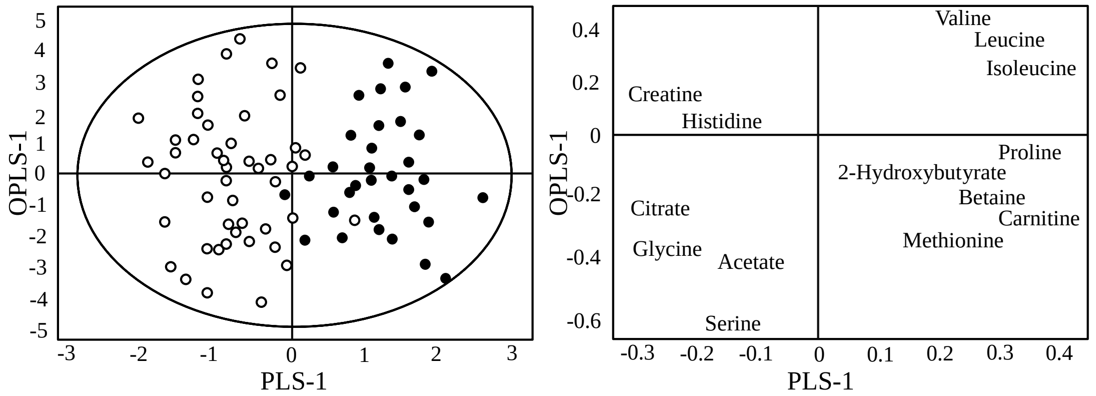

Supplementary Figure S1. Supervised OPLS-DA score scatter plot and loadings plot showing a close to complete separation between men (dots) and women (circles) based on the serum metabolome. Every dot/circle represents one participant. The score scatter plot (left) and loading plot (right) are superimposable and indicate which metabolites that associate with either gender. The model was based on two components, including one orthogonal component (Y-axis), with a R^2 of 0.73 and a Q^2 of 0.61. The distinct clustering of gender indicates that there are considerate differences in the overall metabolome of men and women. The ellipse represents the 95% confidence interval.

Y-variables	Unit	Range (mean)		R^2 / Q^2 values	
Continuous Y-variables		Women (n=47)	Men (n=35)	Women	Men
**Age**	years	30-59 (46)	33-60 (51)	0.57/0.16*	0.75/0.06
**BMI value**	Kg/m^2	16.8-30.3 (23.5)	20.0-33.4 (26.0)	0.25/0.10**	0.41/0.24
**Body fat percentage**	%	14.3-44.9 (30.3)	10.9-34.0 (21.7)	0.37/0.11***	0.49/0.30
**Waist circumference**	cm	64.8-98.7 (82.4)	74.2-155.3 (94.0)	0.34/0.22***	0.37/0.20
**Hip circumference**	cm	81.8-122.0 (99.9)	92.8-111.8 (99.0)	0.45/0.21	N/A
**Waist to hip ratio**		0.73-0.91 (0.82)	0.79-1.04 (0.91)	0.20/0.09***	0.40/0.23
**AEE_{DLW}**	Kcal	489-2974 (1200)	630-3129 (1437)	0.24/0.09	0.27/0.08
**AEE/kg_{DLW}**	Kcal/kg	8.3-49.8 (18.9)	9.0-37.1 (17.3)	N/A	N/A
**Moderate physical activity**	Hours/day	0.05-9.4 (1.6)	0-6.8 (1.8)	N/A	0.34/0.11
**Vigorous physical activity**	Hours/day	0-2.6 (0.5)	0-2.1 (0.6)	0.25/0.14	0.23/0.07
**PAL_{DLW}**	Kcal	1.6-3.6 (2.1)	1.5-3.0 (2.0)	N/A	N/A
Qualitative Y-variables					
**BMI class**		Normal weight= 77% Overweight= 23%	Normal weight= 31% Overweight= 69%	0.42/0.14***	0.57/0.33
**MetS**		0-3 (0) 0= 60% 1= 34% 2= 4% 3= 2%	0-3 (0) 0= 54% 1= 17% 2= 17% 3= 11%	0.19/0.12	0.31/0.12

Supplementary Table S1. All analyzed variables with their respective range of variability and average values as well as the values of explained variance (R^2) and predictive ability (Q^2). Supervised multivariate statistical models were created using partial least square regression (PLS) for the continuous Y-variables and discriminant analysis (DA) were applied for qualitative Y-variables, using PLS-DA when comparing more groups than two (i.e. $MetS_{0-3}$) and orthogonal PLS-DA when comparing two groups (i.e. BMI class or $MetS_{any}$ vs $MetS_{zero}$). N/A indicates when no statistical model

could be created. * $p<0.01$, **$p<0.001$ and *** $p<0.0001$. Abbreviations are as follows in alphabetical order: AEE_{DLW}, activity energy expenditure; BMI, body mass index; MetS, metabolic syndrome; PAL_{DLW}, physical activity level.

	MEN			WOMEN			ALL PARTICIPANTS		
**Serum metabolite**	**Average**	**SD**	**Median**	**Average**	**SD**	**Median**	**Average**	**SD**	**Median**
**2-Aminobutyrate**	9.1	4.1	9.1	7.8	3.1	7.7	8.4	3.6	8.9
**2-Hydroxybutyrate**	16.5	7.6	17.3	13.9	5.3	13.1	15.0	6.5	14.8
2-Hydroxyisovalerate	*3.3*	*1.8*	*3.5*	*3.3*	*1.4*	*3.5*	*3.3*	*1.6*	*3.5*
**3-Hydroxybutyrate**	28.9	42.8	15.5	22.1	18.0	14	25.0	31.1	15
**Acetate**	16.4	4.7	15.7	17.1	5.1	16.1	16.8	4.9	16.1
**Acetoacetate**	12.5	12.7	9.3	9.5	4.9	9	10.8	9.1	9.3
Acetone	*4.2*	*3.4*	*3.2*	*3.9*	*2.6*	*3*	*4.0*	*2.9*	*3.15*
**Alanine**	162.2	29.5	152.9	147.2	31.0	141.5	153.6	31.1	147.2
**Arginine**	42.1	15.5	49.1	42.9	12.0	49.1	42.5	13.5	49.1
**Betaine**	19.9	5.8	19.3	15.7	4.4	15.1	17.5	5.4	16.9
Butyrate	*2.8*	*1.6*	*2.7*	*2.8*	*1.3*	*2.7*	*2.8*	*1.4*	*2.7*
**Carnitine**	18.5	4.6	19.1	13.8	3.1	14	15.8	4.5	15.65
Choline	*4.4*	*1.8*	*4.6*	*4.2*	*0.8*	*4*	*4.3*	*1.3*	*4.25*
**Citrate**	54.9	13.5	56.2	61.4	11.6	60.7	58.6	12.8	58.9
**Creatine**	9.8	4.4	8.4	13.1	5.8	12.1	11.7	5.5	11.4
Creatine phosphate	*5.1*	*2.2*	*4.9*	*4.6*	*1.9*	*4.9*	*4.8*	*2.1*	*4.9*
**Creatinine**	21.0	5.9	21.1	17.9	5.7	17	19.2	6.0	19.05
Dimethyl sulfone	*5.3*	*11.5*	*3.2*	*7.5*	*24.0*	*3.8*	*6.6*	*19.6*	*3.45*
Dimethylamine	*2.0*	*2.2*	*1.3*	*1.8*	*1.8*	*1.3*	*1.9*	*2.0*	*1.3*
Formate	*3.3*	*3.7*	*0*	*3.6*	*4.2*	*0*	*3.4*	*4.0*	*0*
**Glucose**	1986.1	230.9	1982.5	1839.8	219.5	1846.4	1902.2	234.6	1907.9
**Glutamine**	211.8	29.1	212.4	201.3	26.9	203.8	205.8	28.2	211.6
**Glycerol**	889.8	251.8	942.9	880.9	316.5	841.8	884.7	289.0	911.4
**Glycine**	96.1	20.7	93	120.3	44.3	109.8	110.0	37.9	98.65
**Histidine**	26.5	8.1	28.5	28.8	4.7	28.1	27.9	6.4	28.3
**Isoleucine**	27.3	4.1	29.1	21.4	3.3	20.8	23.9	4.7	23.2

**Lactate**	705.3	147.8	697.5	630.8	139.8	583.1	662.6	147.1	644.55
**Leucine**	53.1	7.8	53.9	43.3	5.9	42.3	47.5	8.3	45.7
**Lysine**	56.1	10.5	55.6	50.5	13.5	47.7	52.9	12.5	51
**Methionine**	11.1	3.3	11.3	9.7	2.3	9.8	10.3	2.8	10.45
**Methyl succinate**	6.7	3.2	7	6.2	2.0	5.9	6.4	2.6	6.25
**Myo-inositol**	17.1	9.8	16.1	16.7	7.9	14.3	16.9	8.7	15.25
O-Phosphocholine	*1.7*	*0.8*	*2*	*1.6*	*0.6*	*2*	*1.6*	*0.7*	*2*
**Ornithine**	26.2	7.2	26.8	22.6	6.8	21.8	24.2	7.2	23.9
**Phenylalanine**	25.1	4.9	24.2	24.4	3.9	24.2	24.7	4.4	24.2
**Proline**	86.1	26.2	82.6	64.6	16.2	61.7	73.8	23.5	70.65
**Pyruvate**	18.0	6.3	17.6	18.8	5.9	18.1	18.4	6.0	17.85
**Serine**	50.4	12.8	51.4	53.7	12.6	53.3	52.3	12.7	51.9
**Taurine**	48.8	25.7	52.4	53.5	18.6	55.7	51.5	21.9	54.65
**Threonine**	54.8	21.8	56.8	57.4	20.9	55.3	56.3	21.2	55.7
**Tyrosine**	30.0	6.1	29.2	29.3	5.6	29	29.6	5.8	29.1
**Urea**	273.3	225.1	273.6	229.5	186.1	260.3	248.2	203.5	262.05
**Valine**	103.6	14.7	104.1	86.7	13.1	84.7	93.9	16.0	93.2

Supplementary Table S2. All serum metabolites detected with qualitative profiling of ^{1}H NMR spectra. Metabolites with mean and/or average values below 5uL were excluded in all analysis because their low signal/noise ratio, as indicated in italics. Average and median values as well as standard deviation (SD) are shown for all participants and for men and women separately.

	Body fat percentage	BMI class	MetS
Lower	3-Hydroxybutyrate	Creatinine	Creatinine
	Acetoacetate	Serine	Glutamine
	Serine	3-Hydroxybutyrate	Glycine
	Glycine	Acetoacetate	Serine
	Glutamine		Ornithine
	Urea		Lactate
			3-Hydroxybutyrate
			Carnitine
Higher	Tyrosine	Carnitine	Creatine
	Carnitine	Tyrosine	Myo-inositol
	Pyruvate	Phenylalanine	Urea
	Arginine	Glucose	Methyl succinate
	Phenylalanine	Proline	
	Methyl succinate	Arginine	
	Glucose	Pyruvate	
	Creatine		
	Proline		
	Isoleucine		

	Body fat percentage	Waist circumference	Waist:hip ratio
Lower	Serine	Glycine	Serine
	Glycine	Serine	Glycine
		Urea	Urea
Higher	Pyruvate	Pyruvate	Pyruvate
	Carnitine	Carnitine	Carnitine
	Creatine	Tyrosine	Arginine
	Arginine	Arginine	Tyrosine
	Proline	Methyl succinate	Creatine
	Tyrosine	Creatine	Proline
	Phenylalanine	Valine	Isoleucine
	Methyl succinate	Isoleucine	Valine

	AEE_{DLW}	Moderate physical activity	Vigorous physical activity
Lower		Glycine	Taurine
			Methionine
Higher	Lactate	Taurine	Leucine
	Serine	Carnitine	Glycine
	Phenylalanine	Pyruvate	Creatinine
	Glucose	Lactate	Isoleucine
	Creatinine	Phenylalanine	Valine
	Leucine	Tyrosine	Betaine
	Carnitine	2-Hydroxybutyrate	Serine
	Lysine	Glycerol	Lysine
	Valine	Histidine	Acetate
	Ornithine	Threonine	Arginine
	2-Hydroxybutyrate		
	Histidine		

Supplementary Table S3. Metabolic profiles for men showing all analyzed variables with the most influential (VIP>1) metabolites listed. Negative (lower) and positive correlations (higher) between metabolites and each variable are indicated. No multivariate models could be created for hip circumference, AEE/kg or PAL.

	BMI value	BMI class	MetS	Body fat percentage
Lower	Serine	Serine	Serine	Glycerol
	Methyl succinate	Glycine	Myo-inositol	Serine
	Myo-inositol	Methyl succinate	Creatinine	Myo-inositol
	3-Hydroxybutyrate	Creatinine	Arginine	Creatinine
	2-Hydroxybutyrate	Citrate	Betaine	3-Hydroxybutyrate
	Citrate	2-Aminobutyrate	Acetoacetate	Citrate
		2-Hydroxybutyrate		
		3-Hydroxybutyrate		
Higher	Pyruvate	Glucose	Histidine	Lysine
	Alanine	Carnitine	Lysine	Glycine
	Glucose	Ornithine		Carnitine
	Ornithine	Pyruvate		Creatine
	Creatine	Alanine		
	Carnitine			

	Hip circumference	Waist circumference	Waist:hip ratio
Lower	Serine	Serine	Serine
	Myo-inositol	Myo-inositol	Creatinine
	3-Hydroxybutyrate	3-Hydroxybutyrate	Myo-inositol
		2-Hydroxybutyrate	Methyl succinate
		Methyl succinate	Phenylalanine
			2-Hydroxybutyrate
			Glutamine
			Citrate
			2-Aminobutyrate
			Leucine
Higher	Lysine	Glucose	Urea
	Creatine	Pyruvate	
	Histidine	Carnitine	
	Phenylalanine	Alanine	

	Pyruvate	
	Carnitine	
	Glucose	
	Valine	
	Lactate	
	Glutamine	
	Alanine	

	**AEE**	**Vigorous physical activity**
Lower	Ornithine	Urea
		3-Hydroxybutyrate
		Acetoacetate
Higher	Methyl succinate	Glutamine
	Lysine	Creatine
	Histidine	Phenylalanine
	Creatinine	Carntine
	Carnitine	Lactate
	Acetoacetate	Threonine
	2-Aminobutyrate	Glucose
		Myo-inositol
		Ornithine
		Alanine

Supplementary Table S4. Metabolic profiles for women showing all analyzed variables with the most influential (VIP>1) metabolites listed. Negative (lower) and positive correlations (higher) between metabolites and each variable are indicated. No multivariate models could be created for moderate physical activity, AEE/kg or PAL.

Variable	AEE_{DLW} High Average (SD)	AEE_{DLW} Low Average (SD)	AEE/kg_{DLW} High Average (SD)	AEE/kg_{DLW} Low Average (SD)	PAL_{DLW} High Average (SD)	PAL_{DLW} Low Average (SD)
MetS risk factors	1.0 (0.0)**	1.6 (0.8)			1.1 (0.26)*	1.6 (0.85)
BMI			23.1 (1.4)**	25.8 (3.0)	23.4 (1.6)**	25.9 (3.2)
Body fat percentage			29.4 (4.2)**	36.2 (5.2)	30.3 (4.5)**	36.4 (5.5)
Waist circumference			82.9 (4.9)**	90.1 (6.4)		
Hip circumference			99.9 (3.4)**	105.9 (6.5)	100.8 (3.9)*	105.8 (6.7)

Variable	Moderate physical activity High Average (SD)	Moderate physical activity Low Average (SD)	Vigorous physical activity High Average (SD)	Vigorous physical activity Low Average (SD)
BMI			20.5 (2.0)**	22.8 (1.6)
Body fat percentage			22.5 (5.6)*	27.9 (2.6)
Hip circumference	97.7 (3.8)**	91.3 (5.0)	91.4 (5.4)*	97.6 (3.4)

Supplementary Table S5. Body measures and MetS risk factors that were different between high and low groups of AEE_{DLW}, AEE/kg_{DLW}, PAL_{DLW} as well as moderate and vigorous physical activity in women. Statistical significance is indicated for the respective high compared to the low groups, *$p<0.05$, **$p<0.01$.

Pathway	N(Compounds)	Hits	Raw p	-log(p)	Holm p	FDR	Impact
**Sphingolipid metabolism**	25	1	3.93E-04	7.84E+00	1.96E-02	1.19E-02	0
**Methane metabolism**	34	2	4.75E-04	7.65E+00	2.33E-02	1.19E-02	0.02
**Cyanoamino acid metabolism**	16	3	1.27E-03	6.67E+00	6.07E-02	2.11E-02	0
**Glycine, serine and threonine metabolism**	48	6	1.82E-03	6.31E+00	8.55E-02	2.27E-02	0.44
**Sulfur metabolism**	18	2	3.13E-03	5.77E+00	1.44E-01	3.13E-02	0.03
**Cysteine and methionine metabolism**	56	3	4.68E-03	5.36E+00	2.11E-01	3.90E-02	0.07
**Primary bile acid biosynthesis**	47	2	3.40E-02	3.38E+00	1.00E+00	2.43E-01	0.02
**Porphyrin and chlorophyll metabolism**	104	2	5.07E-02	2.98E+00	1.00E+00	3.17E-01	0
**Aminoacyl-tRNA biosynthesis**	75	13	9.41E-02	2.36E+00	1.00E+00	4.43E-01	0.11
**Glycerolipid metabolism**	32	1	1.11E-01	2.20E+00	1.00E+00	4.43E-01	0.19
**Thiamine metabolism**	24	2	1.14E-01	2.17E+00	1.00E+00	4.43E-01	0
**Glutathione metabolism**	38	2	1.17E-01	2.14E+00	1.00E+00	4.43E-01	0
**Purine metabolism**	92	3	1.19E-01	2.13E+00	1.00E+00	4.43E-01	0.01
**Citrate cycle (TCA cycle)**	20	2	1.33E-01	2.02E+00	1.00E+00	4.43E-01	0.15
**Glyoxylate and dicarboxylate metabolism**	50	2	1.33E-01	2.02E+00	1.00E+00	4.43E-01	0

**Arginine and proline metabolism**	77	8	1.46E-01	1.92E+00	1.00E+00	4.57E-01	0.43
**Nitrogen metabolism**	39	6	2.00E-01	1.61E+00	1.00E+00	5.09E-01	0
**Galactose metabolism**	41		2.03E-01	1.59E+00	1.00E+00	5.09E-01	0
**Lysine degradation**	47	3	2.54E-01	1.37E+00	1.00E+00	5.09E-01	0.15
**Vitamin B6 metabolism**	32	1	2.71E-01	1.31E+00	1.00E+00	5.09E-01	0.02
**Pentose and glucuronate interconversions**	53	1	2.71E-01	1.31E+00	1.00E+00	5.09E-01	0
**Nicotinate and nicotinamide metabolism**	44	1	2.71E-01	1.31E+00	1.00E+00	5.09E-01	0
**Terpenoid backbone biosynthesis**	33	1	2.71E-01	1.31E+00	1.00E+00	5.09E-01	0
**Inositol phosphate metabolism**	39	1	2.82E-01	1.27E+00	1.00E+00	5.09E-01	0.14
**Taurine and hypotaurine metabolism**	20	3	2.82E-01	1.27E+00	1.00E+00	5.09E-01	0.35
**Pyrimidine metabolism**	60	2	2.84E-01	1.26E+00	1.00E+00	5.09E-01	0
**Tyrosine metabolism**	76	3	3.03E-01	1.19E+00	1.00E+00	5.09E-01	0.05
**Synthesis and degradation of ketone bodies**	6	2	3.04E-01	1.19E+00	1.00E+00	5.09E-01	0.7
**Ascorbate and aldarate metabolism**	45	2	3.05E-01	1.19E+00	1.00E+00	5.09E-01	0.02
**Pentose phosphate pathway**	32	2	3.15E-01	1.15E+00	1.00E+00	5.09E-01	0
**Butanoate metabolism**	40	3	3.20E-01	1.14E+00	1.00E+00	5.09E-01	0.13

**Alanine, aspartate and glutamate metabolism**	24	2	3.26E-01	1.12E+00	1.00E+00	5.09E-01	0.21
**Starch and sucrose metabolism**	50	1	3.54E-01	1.04E+00	1.00E+00	5.21E-01	0.02
**Amino sugar and nucleotide sugar metabolism**	88	1	3.54E-01	1.04E+00	1.00E+00	5.21E-01	0
**D-Glutamine and D-glutamate metabolism**	11	1	3.66E-01	1.01E+00	1.00E+00	5.23E-01	0.03
**Pantothenate and CoA biosynthesis**	27	2	4.25E-01	8.55E-01	1.00E+00	5.66E-01	0
**Valine, leucine and isoleucine degradation**	40	3	4.27E-01	8.50E-01	1.00E+00	5.66E-01	0.02
**Valine, leucine and isoleucine biosynthesis**	27	4	4.30E-01	8.43E-01	1.00E+00	5.66E-01	0.05
**Phenylalanine metabolism**	45	3	4.52E-01	7.93E-01	1.00E+00	5.80E-01	0.12
**Phenylalanine, tyrosine and tryptophan biosynthesis**	27	2	5.34E-01	6.27E-01	1.00E+00	6.62E-01	0.01
**Propanoate metabolism**	35	4	5.43E-01	6.11E-01	1.00E+00	6.62E-01	0.03
**Ubiquinone and other terpenoid-quinone biosynthesis**	36	1	5.87E-01	5.32E-01	1.00E+00	6.90E-01	0
**Glycolysis or gluconeogenesis**	31	4	5.93E-01	5.22E-01	1.00E+00	6.90E-01	0.1
**Pyruvate metabolism**	32	3	6.64E-01	4.09E-01	1.00E+00	7.42E-01	0.42
**D-Arginine and D-ornithine metabolism**	8	2	6.68E-01	4.04E-01	1.00E+00	7.42E-01	0
**Histidine metabolism**	44	1	7.47E-01	2.92E-01	1.00E+00	7.94E-01	0.14

**beta-Alanine metabolism**	28	1	7.47E-01	2.92E-01	1.00E+00	7.94E-01	0
**Selenoamino acid metabolism**	22	1	7.84E-01	2.43E-01	1.00E+00	8.17E-01	0
**Lysine biosynthesis**	32	1	8.79E-01	1.29E-01	1.00E+00	8.79E-01	0.1
**Biotin metabolism**	11	1	8.79E-01	1.29E-01	1.00E+00	8.79E-01	0

Supplementary Table S6. Pathway analysis for women comparing $MetS_{any}$ and $MetS_{zero}$ groups. Pathways listed by p-value (Raw p). N(compounds) refers to the number of compounds included in each pathway (library), whereas hits refers to the number of metabolites included in the pathway analysis. Holm adjusted p-values were used for interpretation with significance indicated by p<0.05. False discovery rate (FDR) is also included.
